# Inhibitory effect of *zingiber officinale* towards *Streptococcus mutans* virulence and caries development: *in vitro* and *in vivo* studies

**DOI:** 10.1186/s12866-014-0320-5

**Published:** 2015-01-16

**Authors:** Sadaf Hasan, Mohd Danishuddin, Asad U Khan

**Affiliations:** Medical Microbiology and Molecular Biology lab. Interdisciplinary Biotechnology Unit, Aligarh Muslim University, 202002 Aligarh, India

**Keywords:** *Streptococcus mutans*, qRT- PCR, Biofilm, Dental caries, Gas chromatography-mass spectrometry, Glucosyltransferases, Dental plaque

## Abstract

**Background:**

*Streptococcus mutans* is known as a key causative agent of dental caries. It metabolizes dietary carbohydrate to produce acids which reduce the environmental pH leading to tooth demineralization. The ability of this bacterium to tolerate acids coupled with acid production, allows its effective colonization in the oral cavity leading to the establishment of highly cariogenic plaque. For this reason, *S. mutans* is the only bacterium found in significantly higher numbers than other bacteria in the dental plaque. The aim of this study was to evaluate the effect of crude extract and methanolic fraction of *Z. officinale* against *S. mutans* virulence properties.

**Results:**

We investigated *in vitro* and *in vivo* activity of crude extract and methanolic fraction at sub- MIC levels against cariogenic properties of *S. mutans.* We found that these extracts strongly inhibited a variety of virulence properties which are critical for its pathogenesis. The biofilm formation in *S. mutans* was found to be reduced during critical growth phases. Furthermore, the glucan synthesis and adherence was also found to be inhibited. Nevertheless**,** the insoluble glucan synthesis and sucrose dependent adherence were apparently more reduced as compared to soluble glucan synthesis and sucrose- independent adherence. Biofilm architecture inspected with the help of confocal and scanning electron microscopy, showed dispersion of cells in the treated group as compared to the control. The Quantitative Real Time PCR (qRT-PCR) data had shown the down regulation of the virulence genes, which is believed to be one of the major reasons responsible for the observed reduction in the virulence properties. The incredible reduction of caries development was found in treated group of rats as compared to the untreated group which further validate our *in vitro* data.

**Conclusion:**

The whole study concludes a prospective role of crude extract and methanolic fraction of *Z. officinale* in targeting complete array of cariogenic properties of *S. mutans*, thus reducing its pathogenesis. Hence, it may be strongly proposed as a putative anti- cariogenic agent.

**Electronic supplementary material:**

The online version of this article (doi:10.1186/s12866-014-0320-5) contains supplementary material, which is available to authorized users.

## Background

Dental caries is a complex and multifactorial condition which causes demineralization and progressive destruction of the dental enamel [1]. *Streptococcus mutans*, a member of endogenous oral microflora has long been implicated to play a key role in the pathogenesis of this disease [2,3]. *S. mutans* survival depends strictly on a biofilm lifestyle in its natural ecosystem i.e., dental plaque [4]. Dental plaque formation is important for its persistence since biofilms does not allow easy penetration of chemotherapeutic agents, permitting to cause resistance against antibiotics, immune factors and host-derived antibacterial agents [5]. The ability of this bacterium to produce (acidogenic) and tolerate (aciduric) acids coupled with its property of synthesizing extracellular glucans allows its effective colonization in the oral cavity leading to the establishment of highly cariogenic dental biofilms [6]. Extracellular glucans which are synthesized from sucrose by glucosyltransferases (GTFs) play a critical role in the adhesive interactions of *S. mutans* and contributes to the structural integrity of dental plaque [7]. *S. mutans* expresses three different GTFs viz., GTF B, GTF C and GTF D. The insoluble and soluble glucan is mostly synthesized by GTF B and GTC C respectively. However, GTF C is known to synthesize a mixture of soluble and insoluble glucans [8]. These virulence properties thus provide a unique microenvironment for unobstructed survival of S*. mutans* in the oral cavity [9]. Therefore, approaches to inhibit various factors governing the virulence properties of *S. mutans,* could be an alternative to prevent dental caries.

There are numerous anti-plaque agents that are known to reduce dental biofilm formation amongst which fluoride is a well-known cariostatic agent [10]. However, its excessive use results in side effects like fluorosis and hence its use is limited [11]. Moreover, chlorhexidine which is considered as a standard anti-plaque agent have also been reported as genotoxic [12]. Therefore, despite of the presence of a variety of anti- plaque agents, the quest for an effective agent still continues. Therefore, the development of alternative therapeutic agents with anticariogenic properties and minimal side effects is a promising approach.

Chemotherapeutic agents from natural products have proved to be promising source for the development of new drugs throughout human history [13]. Recently, several studies have shown the feasibility of using medicinal plants as a source of chemotherapeutic agents for the prevention of oral diseases [14].

*Zingiber officinale* (or ginger), is one of the most extensively used herbs in the Indian system of traditional medicine. Many studies have revealed its numerous pharmacological activities, such as, antioxidant, antibacterial, anti-inflammatory, antinociceptive [15], antimutagenic [16] and hepatoprotective [17]. Furthermore, there are several other studies showing the antibacterial effect of *Z. officinale* against a number of micro-organisms including *S. mutans* [18-20]. However, there are hardly any reports evaluating its anticariogenic potential. Hence, in view of the current need of alternative therapeutic approach, we initiated our study to provide an innovative and comprehensive insight of the effect of *Z. officinale* to inhibit various virulence traits of *S. mutans*.

## Methods

### Ethics statement

This research methodology was conducted in accordance with institutional ethical standards. The study on animals was approved by “Interdisciplinary Biotechnology Unit, Institutional Ethical Committee”.

### Plant material and preparation of extracts

Dried roots of *Zingiber officinale* were purchased from the local market of Aligarh and the species was identified and authenticated in Department of Botany, A.M.U., Aligarh, India. Crude and solvent fractions of the rhizome were prepared as described earlier [3].

### Bacterial strain and culture medium

Bacterial strain used in this study was *S. mutans* UA159, which was grown in Brain Heart Infusion (BHI) Broth (Himedia Labs, Mumbai, India) at 37°C in a 5% CO_2_ anaerobic atmosphere. It was sub cultured regularly and stored at-80°C as glycerol stock.

### Determination of bacteriostatic (MIC) and bactericidal (MBC) concentration

The MIC and MBC of the crude (CR) extract and methanolic (ME) fraction against *S. mutans* was determined by microdilution method [21]. For the determination of MIC, twofold serial dilution of the compound was performed. The inoculum was prepared in BHI, and density was adjusted to 0.5 Mcfarland standards (10^8^ CFU ml^−1^) and diluted to 1: 100 for the broth microdilution procedure. Microtitre plates were incubated at 37°C, and the MIC was recorded after 24 h. The concentrations ranged from 2.44 to 5000 μg ml^−1^ in a series of twofold dilutions. The MIC was determined as the lowest concentration that totally inhibits visible bacterial growth while MBC was determined by subculturing the test dilutions on a mitis salivarius (MS) agar plates and incubated further for 24 h. The highest dilution that yielded no bacterial growth on solid medium was taken as MBC. All these determinations represent the mean of three independent experiments.

### Gas chromatography-mass spectrometry (GC-MS) analyses of crude extract and methanolic fraction of *Z. officinale*

Constituents of CR extract and ME fraction were analyzed by GCMS-QP_2010_ plus using the protocol described elsewhere [3]. The major constituents of the crude and ethanolic fractions were identified by drawing a comparison between the GC retention times with MS reference database of NIST05s.LiB.

### Kinetic killing assay

The kinetics of the bacterial-killing effect of CR extract and ME fraction of *Z. officinale* was assessed against *S. mutans* by using modified method of Koo *et al.* [22]. Tubes containing *S. mutans* suspension (5 × 10^7^ CFU ml^−1^) and different sub- MIC levels of crude extract and methanolic fraction (8, 16, 32, 64 and 128 μg ml^−1^, final concentration) were incubated. Samples were removed for colony counting at different time interval over 24 h. These samples were serially diluted with PBS and aliquots of 5 μL were incubated into BHI agar. The plates were then incubated, and CFUs were counted. A bactericidal effect was defined as a > 3-log CFU ml^−1^ decrease from the original inoculum.

### Sucrose-dependent and sucrose-independent adherence assay

Adherence to glass surface assay was performed by the method of Hamada *et al.* with slight modifications [23]. Briefly, the bacteria were grown at 37°C at an angle of 30° for 24 h in a glass tube containing 10 ml of BHI with or without 5% (w/v) sucrose and sub-MIC concentrations of the extracts (CR and ME). The solvent controls included BHI with (sucrose dependent) and without sucrose (sucrose independent) and equivalent amounts of DMSO and ethanol. After incubation, the planktonic cells were decanted gently from the glass tubes. The adhered cells were then removed by adding 0.5 M of NaOH followed by vortexing. The cells were washed and suspended in saline. The adherence was quantified spectrophotometrically at 600 nm. All these determinations were performed in triplicates, using untreated BHI medium as control.

$$ \mathrm{Percentage}\ \mathrm{adherence} = \left(\mathrm{O}.\mathrm{D}.\ \mathrm{of}\ \mathrm{adhered}\ \mathrm{cells}/\mathrm{O}.\mathrm{D}.\ \mathrm{of}\ \mathrm{total}\ \mathrm{cells}\right) \times 100. $$

### Biofilm formation assay

The inhibitory effect of CR extract and ME fraction of *Z. officinale* on biofilm formation by *S. mutans* was performed using the protocol described elsewhere [24]. Briefly, 50 μL of overnight culture of *S. mutans* (10^5^–10^6^ CFU ml^−1^) was inoculated into 150 μL of BHI with 5% (w/v) sucrose containing various concentrations of CR extract and ME fraction with respective controls. After incubation at 37°C for 24 h, media and unbound cells were decanted from the microtitre plates. The remaining planktonic cells were removed by gently rinsing with sterile water. The wells with attached cells (biofilms) were fixed with formalin (37%, diluted 1:10) plus 2% sodium acetate. Each well was stained with 200 μL of 0.1% Crystal Violet for 15 min at room temperature. After two rinses with sterile water, bound dye was removed from the cells with 100 μL of 95% alcohol. Plates were then set on a shaker for 10 min to allow full release of the dye. Biofilm formation was then quantified by measuring optical density of the suspension at 600 nm by a microplate reader (BIORAD iMark TM Microplate reader, India). Separate biofilms were formed in the presence extracts for time-dependent effect at 6, 12, 20 and 24 h.

### Inhibition of water-insoluble and soluble glucan synthesis

To examine the inhibitory effect on water insoluble and soluble glucan production by *S. mutans*, sub-MIC concentration CR extract and ME fraction of *Z. officinale* was added to the inoculum in BHI containing 5% sucrose and incubated at 37°C. Crude GTFs were prepared from the culture supernatants of *S. mutans* by previously described method [25]. A reaction mixture consisting of 0.25 ml of crude enzyme and varying concentrations of the extracts (CR extract and ME fraction) in 20 mM phosphate buffer (pH 6.8) containing 0.25 ml of 0.4 M sucrose was incubated at 37°C for 18 h. The tube was removed post incubation and the contents were washed with distilled water. Total amounts of water-soluble and insoluble glucan were measured by the phenol-sulphuric acid method [26]. Three replicates were made for each concentration of the extracts and ethanol was used as control.

### Effect on cell-surface hydrophobicity of *S. mutans*

The cell surface hydrophobicity of *S. mutans* was measured according to Microbial adhesion test to hydrocarbon [27]. Briefly, cells grown in BHI medium supplemented with different sub- MIC concentrations of the extracts. These cells were washed thrice and suspended in 0.85% sterile saline so that their optical density was adjusted to 0.3 at 600 nm. The cell suspension (3.0 ml) was placed in tubes and 0.25 ml of toluene was added. The tubes were agitated uniformly in a vortex mixer for 2-3 min and allowed to equilibrate at room temperature for 10 min. After toluene phase separation from the aqueous phase, the O.D. of the aqueous phase was determined spectrophotometrically at 600 nm. *S. mutans* with a hydrophobic index >70% was arbitrarily classified as hydrophobic.

### Effect on glycolytic pH drop

The level of the glycolytic pH drop of *S. mutans* was measured, as described elsewhere [28]. Briefly, *S. mutans* cells from the suspension cultures were harvested and washed once with salt solution containing 50 mM KCl and 1 mM MgCl_2_. They were suspended in a salt solution containing sub- MIC concentration (128 μg ml^−1^) of the extracts (CR extract and ME fraction) or the vehicle control. The pH was adjusted between 7.2-7.4 with 0.2 M KOH solution. Sufficient glucose was added to obtain a concentration of 1% (w/v) and the decrease in pH was assessed over a period of 60 min using a glass electrode. The initial rate of pH drop, was calculated using the pH values in the linear portion (0-10 min) which can give the best measure of the acid production capacity of the cells.

### Cell permeabilization and F- ATPase activity

Permeabilization of *S. mutans* cells was done by subjecting the cells to 10% toluene (v/v) followed by two cycles of freezing and thawing as described elsewhere [25]. F-ATPase was measured in terms of the release of phosphate. The standard reaction mixture consisted: 75 mmol of Tris-maleate buffer (pH 7.0) containing 5 mM ATP, 10 mmol of MgCl_2_, permeabilized cells and different concentrations of the crude extract and the methanolic fraction (8–128 μg ml^−1^). The released phosphate was determined over 10 min of reaction time by the method of Bencini *et al.* [29].

### Effect on Surface Protein Ag I/ II

The total protein from *S. mutans* cells was conjugated to rabbit anti-Ag I/II to equate and calculate the levels of Ag I/II protein (or SpaP). The amount of protein Ag I/II from control and treated samples was calculated. 10 μg of total protein from the treated and untreated samples was dissolved in 100 μl of 20 mM carbonate buffer (pH 9.3) and was coated on the polystyrene plates. The plates were washed with PBS-Tween and was blocked with 5% skimmed milk in bicarbonate buffer. The plates were washed again with PBS-T thrice and then incubated with rabbit polyclonal Ag I/II antibody at 37°C for 2 h. The plates were rewashed thrice with PBS and the incubated for 2 h with 100 μl of anti-rabbit peroxidase coated antibody, dilutions ranging from 1:100 to 1:1000000. The plates were washed again with PBS and the 50 μl of TMB (3, 3′, 5, 5′- tetramethylbenzidine). The reaction was stopped immediately after the color appeared using 50 μl of 4 N H_2_SO_4_ [3]. Quantification of the intensity was done by measuring optical density (OD) at 450 nm by a microplate reader (BIORAD iMark TM Microplate reader, India).

### Target preparation and docking analyses

The crystal structure of C-terminal region of Surface Protein Antigen (SpaP or Ag I/ II) of *S. mutans* was downloaded from protein databank having PDB ID: 3QE5 whereas, the structure of BrpA (Biofilm Regulatory Protein) was modelled using Modeller 9v7 [30]. The target preparation for docking was done by removing all waters molecules from the structure and by adding hydrogen atom to the target protein (SpaP or BrpA). Program Q-Site Finder was used for the detection of active site of the protein [31] (Laurie and Jackson 2005). Two dimensional structures of selected compounds were downloaded from Pubchem database. GOLD 5.0 version was used to study the binding orientation of selected compounds into the *S. mutans* SpaP and BrpA structure [32]. The default parameters of the automatic settings were used to set the genetic algorithm parameters. The best protein-ligands complexes were selected based on the scoring function of GOLD fitness score.

### Confocal microscopy

To analyse the effect of the CR extract and ME fraction on *S. mutans* biofilm, cells were grown on glass coverslips. Sample preparation was done using the protocol described elsewhere [3]. Fluorescence emission was observed using confocal scanning laser microscope (Fluoview FV200). The images of control and treated samples were averaged and compared.

### Scanning electron microscopy

The effect of the plant extracts (CR and ME) on structural integrity of biofilm was also observed by scanning electron microscopy (SEM). The samples were prepared for SEM examination as described elsewhere [25]. Also, the aseptically removed jaws of the animals (wistar rats) were stored in normal saline and were directly visualized under SEM. The experiment was run in triplicates. Samples were analysed by SEM (Hitachi S-3000 N; High Technology Operation, Japan) at several magnifications.

### RNA isolation and real-time quantitative PCR (qRT-PCR)

To analyse the effect of plant extracts (CR and ME) on expression of virulence genes of S*. mutans*, the organism was cultured in BHI medium supplemented with sub MIC concentration of the extracts. Bacterial culture (OD_600_ = 0.8) were diluted at a ratio of 1:50 followed by their inoculation into BHI media and were incubated at 37°C for an overnight growth. RNA was isolated and purified and qRT- PCR was performed using the protocol described elsewhere [25]. The primer sequences are provided in Additional file 1.

### *In vivo* toxicity studies of CR fraction and ME fraction

Acute oral toxicity of plant extracts was evaluated in accordance to Organization for Economic and Cooperation Development (OECD) guidelines (2001) for testing plant extracts. A limit test (5000 mg kg^−1^ body weight of animal) was carried out using five male wistar rats ranging from 80-120g in weight, in all the groups used. All animals were observed for change in weight, behavioural changes and mortality till 14^th^ day post administration of dose.

### Caries induction in rats

To determine the effects of the CR extract and ME fraction on oral colonization and cariogenic potential of *S. mutans*, a total of 30 rats were purchased. These animals were divided three groups; a control and two test groups (n = 10 per group). All the animals were fed with erythromycin water (100 μg ml^−1^) and a regular diet for 3 days in order to lower the microbial load. To confirm the absence of *S. mutans* colonization in the oral cavity, oral swab was plated on MSB plates. The animals were offered 5% sucrose diet ad libitum throughout the experiment in order to enhance the infection by *S. mutans.* On 4^th^ day, the animals were inoculated with 1.4 × 10^10^ CFU of streptomycin resistant strain of *S. mutans* (MT8148R), onto the animals’ molars surfaces once every day for five consecutive days to allow oral colonization. The treatment was given twice daily, by applying the plant extracts topically on the teeth of animals by means of a camel’s hair brush. Swab samples were then taken from the surfaces of animal molars on the first day and at the first, third, sixth, eighth and tenth week’s post-inoculation. The samples from control and treated groups were pooled in 2 ml 10 mM potassium phosphate buffer, serially diluted and plated on MSB agar plates containing streptomycin for total cell counts. The plates were incubated at 37°C for 2 days before enumeration of colonies of *S. mutans*. The percentages of the *S. mutans* cells were calculated to determine its oral colonization in the animals. At the end of the experimental period, all the animals were sacrificed. The jaws were then aseptically dissected and sonicated in 5 ml of 154 mM sterile NaCl in order to dislodge the dental plaque. These samples of plaque were serially diluted and were streaked on mitis salivarius agar plates to estimate the *S. mutans* population. These plates were incubated at 37°C for 2 days before enumeration of colonies. All of the jaws were defleshed, and suspended in 3.7% formaldehyde until caries scoring. All molars of the animals were examined under a dissecting microscope and carious lesions were scored by a Larson’s modification of Keyes system [33].

The results obtained were analysed by Student’s t test, with p < 0.05 considered as statistically significant.

### Statistical analysis

The data are presented as mean ± standard deviation. The intergroup differences were estimated by one-way analysis of variance (ANOVA), followed by a post hoc multiple comparison (Tukey’s test) to compare the multiple means. Values were considered statistically significant when *p* value was <0.05. The statistical analyses were performed using SPSS 12 software.

## Results

### Minimum inhibitory concentration (MIC) and minimum bactericidal concentration (MBC)

The MIC and MBC of the both the plant extracts (CR and ME) of *Z. officinale* against *S. mutans* was found to be 256 μg ml^−1^ each.

### Phytochemical analysis of the extracts by GC- MS

The phytochemical composition of CR extract and ME fraction of *Z. officinale* determined by GC- MS are listed in detail in Additional file 2. Some of the secondary metabolites like D-nerolidol, α Curcumen, Tran- 10 – Shogaol, Paradol etc. were found to be common in both the extracts.

### Kinetic killing assay

**Figure 1:**
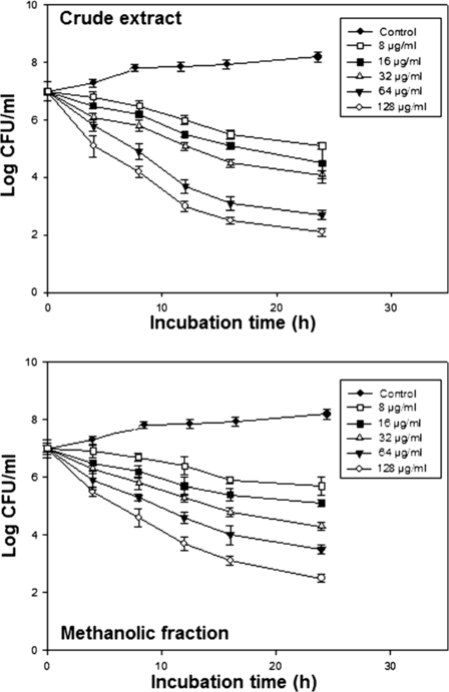
**Kinetic killing curve.** The bacteriostatic activity of CR extract and ME fraction at different sub- MIC levels on *S. mutans* cell cultures over 24h of incubation.

The kinetics of the antimicrobial effect of CR extract and ME fraction against *S. mutans* is demonstrated in Figure 1. Both these extracts killed *S. mutans* in a time as well as dose dependent manner. CR extract at 128 μg ml^−1^ (0.5 MIC) showed an antibacterial activity against *S. mutans*, with more than 5-log CFU ml^−1^ decrease after 24 h of incubation, whereas ME fraction at the same concentration showed a reduction of 4- log CFU ml^−1^.

### Inhibitory effect on bacterial adherence

**Figure 2:**
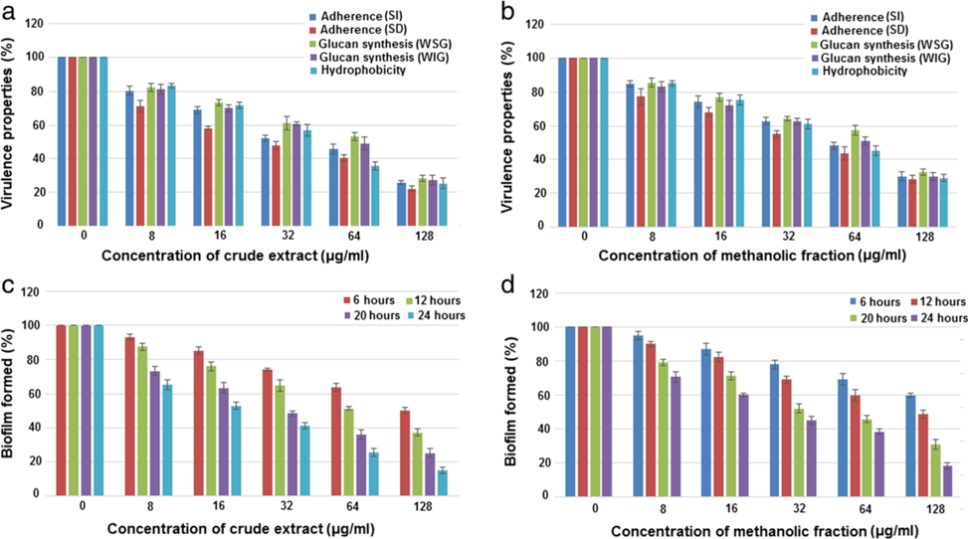
**Effect on adherence, glucan synthesis, hydrophobicity & biofilm formation.** Inhibitory effect of CR extract and ME fraction on the glass-dependent adherence in the absence (sucrose-independent SI) and presence of 5% sucrose (sucrose-dependent SD), water- soluble (WSG) and insoluble glucan (WIG) synthesis and on surface hydrophobicity by *S.mutans* **(a & b)**. Also their effect on biofilm formation at 3h, 6h, 12h and 24 h of incubation **(c & d)**. Each value is an average of triplicate assays, and each bar indicates ± standard deviation (n = 3).

The effect on sucrose dependent (SD) and sucrose independent (SI) adherence of *S. mutans* treated with different concentrations of the CR extract and ME fraction is given in Figure 2 (a and b). Both of them displayed a strong anti-adherence activity by inhibiting the SD as well as SI adherence. However, the inhibition in SD adherence was slightly pronounced compared to SI adherence. CR extract at 128 μg ml^−1^ was found to reduce SD and SI adherence by 78% and 73% respectively, whereas ME fraction at the same concentration reduced the same by 72% and 70% respectively.

### Inhibition of glucosyltransferases activity

The effect of different concentrations of CR extract and ME fraction was assessed against GTF activity in terms of the synthesis of water soluble glucan (WSG) and water insoluble glucan (WIG). The inhibition of glucan synthesis was found to be concentration dependent in the reaction. However, the reduction was observed to be more significant in case of water-insoluble glucan. At sub- MIC level (128 μg ml^−1^), the CR extract reduced the formation of WSG and WIG to approximately > 70% as shown in Figure 2 (a & b). However, ME fraction was found to reduce the same by approximately 70%.

### Effect on cell-surface hydrophobicity index of *S. mutans*

The hydrophobicity index (HPBI) was significantly reduced in a dose dependent manner when compared with control. As shown, at sub-MIC concentration (128 μg ml^−1^), the CR extract and ME fraction reduced the HPBI by 75% and 71% respectively (Figure 2a & 2b).

### Biofilm formation by *S. mutans*

The CR extract and ME fraction inhibited biofilm formation in a concentration dependent manner. The effect was tested at 6, 12, 20 and 24 h to evaluate whether it could affect *S. mutans* in each phase of biofilm growth. The results demonstrated that the effect of both the extracts was concentration-dependent as well as dependent on biofilm growth phases as shown in Figure 2 (c & d). The percentage of adherent cells under various concentrations of extracts was found to decline with every successive growth phase. At 6 h of biofilm growth, CR extract and ME fraction at 128 μg ml^−1^ reduced biofilm formation by 50% and 40% respectively. At 12 h, 50% reduction was observed by CR extract and ME fraction at a concentration of 64 μg ml^−1^ and 128 μg ml^−1^ respectively. However, at 20 h and 24 h, the percentage of adherent cells were reduced to >70% and > 80% at maximum concentration as compared to control group. Precisely, biofilm formation was inhibited mostly during the active accumulated phase, initial plateau accumulated phase and plateau accumulated phase of growth.

### Glycolytic pH drop

**Figure 3:**
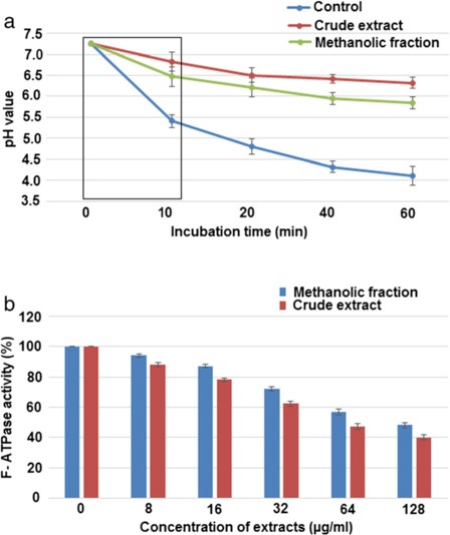
**Inhibitory effect on acid production and adaptation.** Effect of sub-MIC concentration (128 μg ml^−1^) of CR extract and ME fraction on **(a)** glycolytic pH-drop (the values enclosed in box corresponds to the initial rate of the pH drop) **(b)** F- ATPase activity.

As shown in Figure 3a, the glycolytic acid production of *S. mutans* was significantly inhibited by CR extract and ME fraction at a concentration of 128 μg ml^−1^ (0.5 MIC). In control, the onset pH 7.25 was decreased to 4.11 after 60 min of incubation. Whereas, after treatment with ME fraction and CR extract this acidic pH (4.11) was raised to 5.85 and 6.32 respectively. Above all, the pH drop recorded in first 10 min of incubation (known as initial pH drop) was observed maximum in case of control with pH values of 7.25 to 5.41. However, CR extract showed minimum pH drop with values 7.25 to 6.83. (Tukey test, p < 0.05).

### F- ATPase activity

The result of F- ATPase activity assay is shown in Figure 3b. It was found that the F-ATPase activity of *S. mutans* cells was inhibited dose-dependently. At 64 μg ml^−1^ (0.25 MIC), ME fraction and CR extract declined the F- ATPase activity by 44% and 53% respectively. However, at 128 μg ml^−1^ (0.5 MIC), the activity was reduced to 52% and 60% respectively as compared to the control (p < 0.05).

### Inhibitory effect on surface protein antigen, SpaP

**Figure 4:**
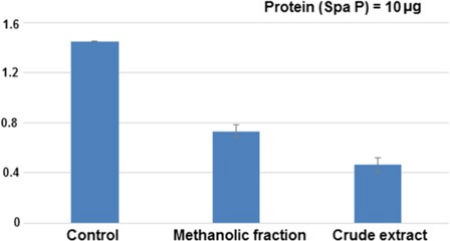
**Effect on surface protein antigen.** Direct binding ELISA of total protein from untreated *S. mutans* (control) and *S. mutans* treated with sub- MIC concentration of CR extract and ME fraction against polyclonal antibodies of Ag I/II rose in rabbit. Data are means ± SD (n = 3).

As shown in Figure 4, the reduction of surface protein antigen (spaP or Ag I/II) in samples treated with ME fraction and CR extract was found to reduce by approx. 50% (OD 0.73) and 70% (OD 0.46) respectively at 1:100 antibody dilution as compared to the control (OD 1.45).

### Docking studies on spaP and brpA with different compounds of CR extract and ME fraction

**Figure 5:**
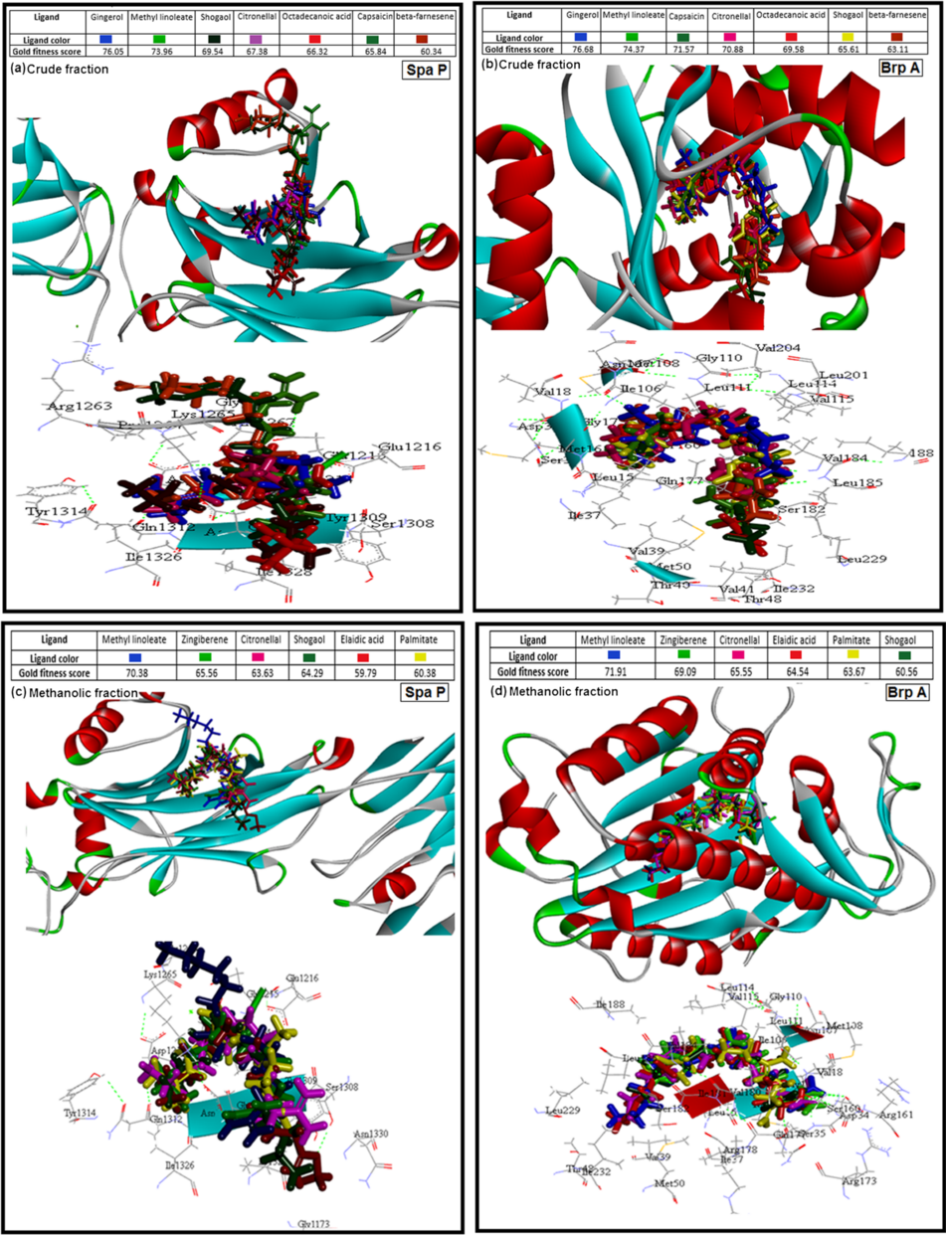
**Docking analysis. **Binding pattern of the compounds from crude extract **(a** **&** **b)** and methanolic fraction **(c** **&** **d)** analysed by GC-MS showing best docking score within the active sites of Spa and Brp A.

All the ligands depicted by GC- MS were docked into the active sites of proteins SpaP and BrpA. Of all these ligands, seven ligands from crude extract (Gingerol, Methyl linoleate, Shogaol, Citronellal, Octadecanoic acid, Capsaicin and Beta- farnesene) and six from methanolic fraction (Methyl linoleate, Zingiberene, citronellal, Elaidic acid, Palmitate and Shogaol) were identified as the best compounds depending on their gold score as shown in Figure 5. Compounds from crude extract (Figure 5a & 5b) were found to dock in the active site of target proteins SpaP and BrpA with a range of 60-76 and 63-76 of Gold fitness score respectively. Whereas, compounds from methanolic fraction (Figure 5c & 5d) were found to interact with a range of 60-71 Gold fitness score for both the proteins. Furthermore, in case of crude extract, thirteen amino acids (Glu1215, Glu1216, Gly1261, Arg1263, Pro1264, Lys1265, Ala1267, Ser1308, Tyr1309, Gln1312, Tyr1314, Ile1326 and Ile1328) were found to be common for all the compounds in stabilizing the complex of SpaP protein as shown in Figure 5a and twenty three amino acids (Leu15, Met16, Gly17, Val18, Ile37, Val39, Thr48, Met50, Ile106, Met108, Gly110, Leu111, Leu114, Val115, Val118, Gln177, Ser182, Val184, Leu185, Ile188, Leu201, Val204 and Leu229) were found to be common for all the compounds in stabilizing the complex of BrpA protein as shown in Figure 5b. Whereas, in case of methanolic fraction, eleven amino acids (Gly1173, Glu1215, Glu1216, Pro1264, Lys1265, Ser1308, Tyr1309, Gln1312, Tyr1314, Ileu1326 and Asn1330) were found to be common for all the compounds in stabilizing the complex of SpaP protein as shown in Figure 5c and twenty three amino acids (Leu15, Gln17, Val18, Asp34, Ser35, Ile37, Val39, Ile106, Asn107, Met108, Gly110, Leu111, Leu114, Val115, Ser160, Arg161, Arg173, Arg178, Val180, Ser182, Ileu188, Leu229 and Ile232) were found to be common for all the compounds in stabilizing the complex of BrpA protein as shown in Figure 5d.

### Impairment of biofilm formation visualized by scanning and confocal electron microscopy

**Figure 6:**
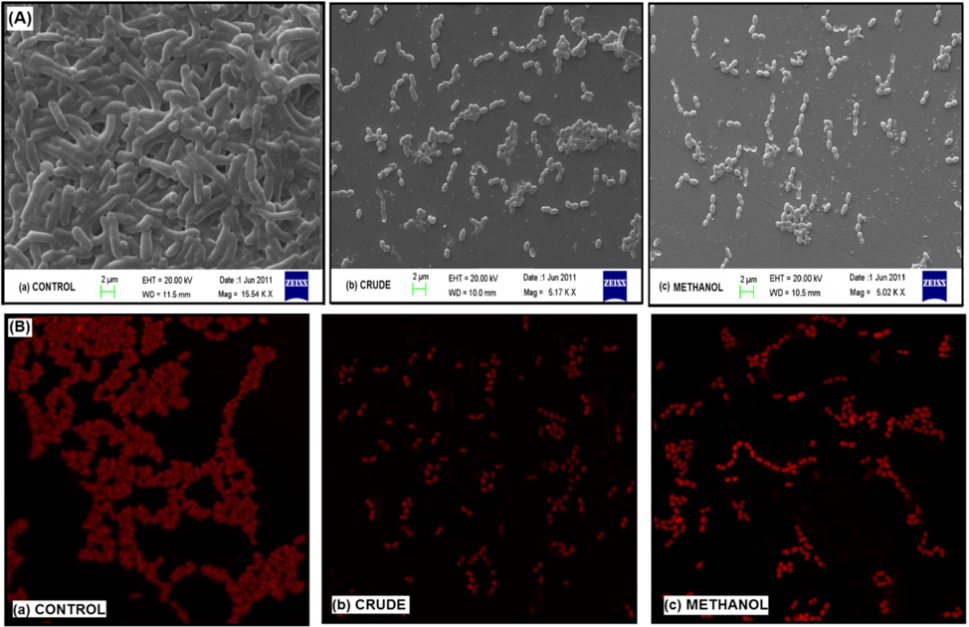
**Electron microscopy.** SEM **(A)** and CLSM images **(B)** of S. mutans biofilm formed in the presence and absence of the sub- MIC levels of extracts after 24 h of incubation.

SEM depicted the impact of CR extract and ME fraction on the activity of *S. mutans* to synthesize glucans and on the structural integrity of biofilm formed (Figure 6A). In concordance with the aforesaid results the treated group displayed significant dispersion of cells suggesting reduced amount of glucan synthesis that would have otherwise resulted in adherence. On the contrary, the control sample as shown in Figure 6A (a) showed apparent clumping of cells with chain formation embedded into the exopolysaccharide pool.

However, the distortion of biofilm architecture of *S. mutans* was also analysed by confocal laser scanning microscopy (CLSM). As shown in Figure 6B, in the absence of treatment (a), the cells were aggregated with an evident chain forming pattern. However, unlike control, the groups treated showed apparent scattering of cells without any cluster or chain formation, suggesting reduced interaction between cells, resulting in impaired biofilm formation. However, the cells treated with CR extract were more dispersed as compared to the ones treated with ME fraction.

### Gene expression profile

**Figure 7:**
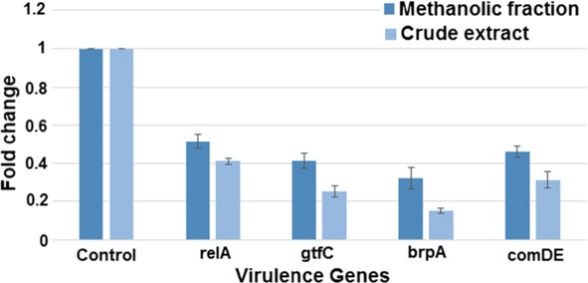
**Relative quantification of selected virulent genes expression by quantitative RT-PCR.** Expression profile by real time PCR of various virulence genes of *S. mutans* in response to the treatment with sub- MIC levels of extracts. Each value is an average of triplicate assays. Data are means ± SD (n = 3).

The expression profile of different virulence genes (*relA, gtfC, brpA* and *comDE*) of *S. mutans* treated crude extract and methanolic fraction was determined (Figure 7). The entire set of virulence genes was found to be down regulated after treatment. Crude extract repressed the expression level of *relA, gtfC, brpA* and *comDE* by 60%, 74.6%, 85% and 68.6% respectively as compared to the control. Likewise, methanolic fraction suppressed the expression level of *relA, gtfC, brpA* and *comDE* by 48.5%, 59%, 68% and 54% 54% respectively. Evidently, both crude extract and methanolic fraction suppressed *brpA* gene to the maximum (85% and 68% respectively) followed by the suppression of *gtfC* gene.

### Caries reduction *in vivo*

**Figure 8:**
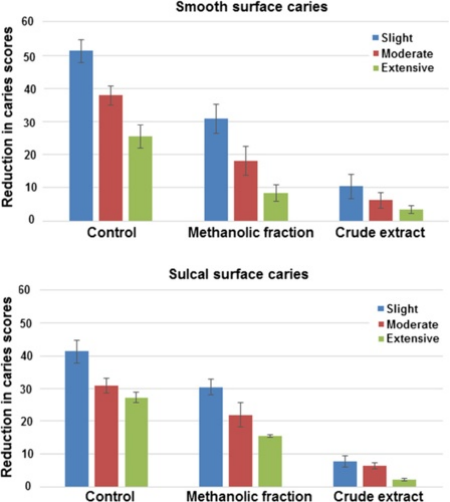
**In vivo validation.** Effect of sub- MIC level of crude extract and methanolic fraction of *Z. officinale* on dental caries development in rats; means (SE), Keyes’ score.

After toxicity assays were performed, the animals did not display any behavioural or weight changes, neither any mortality occurred post oral toxicity assay indicating that CR extracts and ME fraction were absolutely non- toxic. The weekly recovery of *S. mutans* cells over 5 weeks post treatment is shown in Additional file 3. It was found that there was significant reduction in the recovery of *S. mutans* from rats treated with CR extract and ME fraction. However, CR extract showed slightly better activity than ME fraction. Furthermore, Figure 8 shows the reduction in smooth as well as sulcal surface caries after treatment. The overall reduction in smooth surface caries was more pronounced as compared to the sulcal surface caries post treatment. CR extract consistently showed better anticariogenic activity by reducing severity of smooth surface caries to 80% (slight), 83% (moderate) and 86% (extensive) as compared to ME fraction which reduced the caries by 40% (slight), 52% (moderate) and 66% (extensive).

### Scanning electron micrograph and radiographs of untreated and treated rats teeth

**Figure 9:**
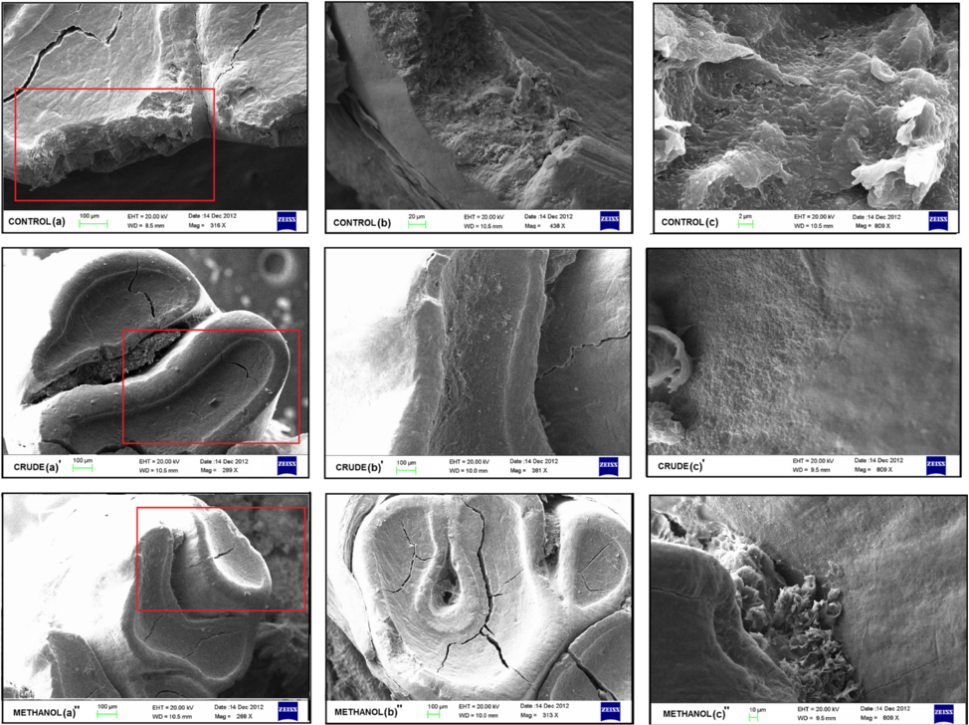
**Scanning electron micrographs of aseptically removed rat teeth.** SEM analysis of rats teeth to evaluate the effect of CR extract and ME fraction on development of caries and extent of demineralization in untreated **(a)** and groups treated with crude extract and methanolic fraction **(b’ & b”)**. Panels **c**, **c**’ & **c**” show dental surface of the teeth to observe biofilm formation. Panel **b**, **b**’ and **b**” shows the zoomed area focussed in red square of panel **a**, **a**’ and **a**” respectively.

The SEM analysis of the rats’ teeth clearly depicted the demineralization of the dental margins in untreated group (Figure 9a), while the groups treated with crude extract and methanolic fraction showed smooth dental margins as shown in Figure 9b’ and 9b’ respectively. Furthermore, the panels in Figure 9 c, c’ c” shows the dental surface. It was clearly observed that in untreated samples (Figure 9c), the surface has an evident biofilm embedded in exopolysaccharide pool whereas in treated groups (Figure 9c’ & 9c”), the dental surface was clear from any such exopolysaccharide projections as visible in control.

**Figure 10:**
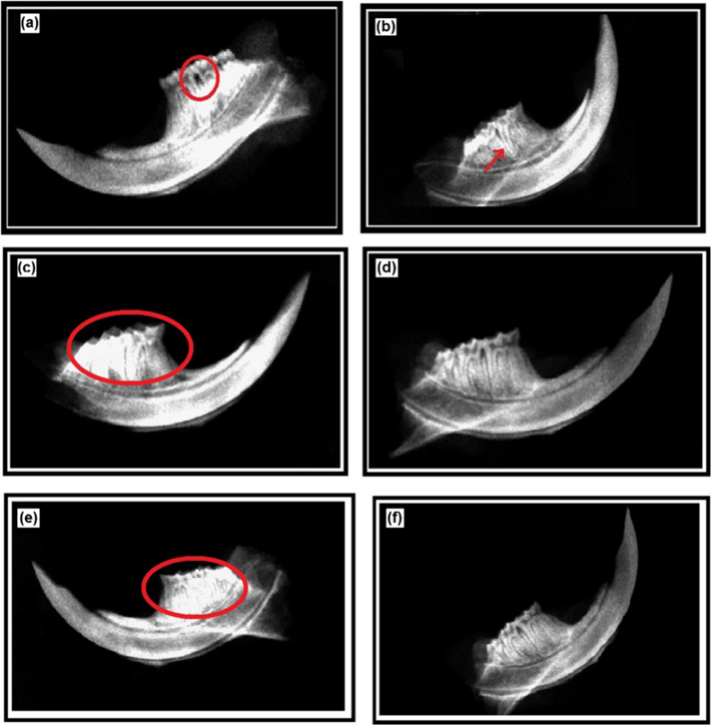
**Digital radiographs of rat teeth.** Digital radiographs of untreated **(a & b)** and treated rat teeth with CR extract **(c & d)** and methanolic fraction **(e & f)** rat teeth to evaluate the anticariogenic effect of CR extract and ME fraction.

Figure 10 shows the digital radiographs of untreated (a & b) and treated rat teeth with CR extract (c & d) and ME fraction (e & f). As shown in Figure 10a, there was an evident black space (as spot) that corresponded to a carious lesion whereas, 10b was the flipped side of the jaw showing the caries lesion reaching to the pulp cavity revealed by a V- lining surrounding the bottom of the tooth. This concavity reaching to the pulp of the tooth corresponded to an inflamed and infective condition known as pulpitis. However, in 10c and 10e, there was no such space or spot seen that confirmed the absence of caries. Furthermore, 9d and 9f show normal radiograph without any sign of pulpitis which could have been sequelae of dental caries. The line extending downwards are the roots of the teeth and should not be confused with inflammation of the pulp, as they are not surrounding the root of the tooth in a V- shape pattern. All these radiographs were confirmed by an expert of the field.

## Discussion

This study offers *in vitro* and *in vivo* experimental evidences that the CR extract and ME fraction of *Z. officinale* show exceptional anticariogenic potential. The GC- MS analysis of these extracts showed that there are numerous phytochemicals that are common in CR extract and ME fraction, which could be accounted for ME fraction activity being comparable to that of CR extract.

From the results obtained, the MIC value of CR extract and ME fraction against *S. mutans* was quite significant [34]. Also, in kinetic killing assay, both these extracts resulted in a rapid decrease of bacterial viable counts even at low concentrations suggesting that these extracts can be delivered in low but effective concentrations in the oral cavity to inhibit dental caries.

Furthermore, it is well documented that adherence is one of the most important step in establishment of dental caries and its inhibition could be a strong step against the impairment of its virulence [35]. The sucrose-independent (SI) adherence is usually because of hydrophobic interactions between the cells and the adhering surface. As per the results obtained, there is reduction in SI adherence as well as hydrophobicity. Because of the reduced hydrophobic interactions between the cells and the surface, bacteria were unable to adhere. Thus, the reduction in hydrophobicity could be directly co- related with the reduction in SI adherence. Sucrose dependent adherence, on the other hand, is mediated via synthesis of glucans. GTFase is the enzyme responsible for the conversion of sucrose to sticky glucans, which promotes the adherence of *S. mutans* to the surface of the tooth. Hence, SD adherence depends upon glucan production. It is evident from the results that CR extract and ME fraction reduced the glucan synthesis in addition to SD adherence, as flavonoids are known to have anti- GTFase activity [36]. As higher amount of insoluble glucans in the matrix is directly associated with increased cariogenicity in humans [3], the inhibition he synthesis of water insoluble glucan (as per the results obtained) will lead to an altered exopolysaccharide matrix resulting in ineffective adherence. Another important event in pathogenesis of *S. mutans* is the formation of biofilm which is also mediated by glucans. The reduction in water insoluble glucan (WIG) will result in a considerable reduction of biofilm formation as it will interfere with the pathogenesis by disrupting physical integrity and stability, reducing the availability of binding sites for *S. mutans* [37]. As per the results obtained, CR extract and ME fraction reduced biofilm formation during different growth phases i.e., in initial attachment phase (6 h), active accumulated phase (12 h), initial plateau accumulated phase (20 h) and plateau accumulated phase (24 h), suggesting not only the inhibition of biofilm formation but also its maturation at critical developmental stages. Hence, based on these results, it is apparent that CR extract and ME fraction could inhibit the GTF activity of *S. mutans* and thereby the bacterial adherence, signifying their potential to modulate the development and accumulation of *S. mutans* dental biofilms (monospecies).

The results obtained from glycolytic pH drop showed inhibitory activity of the extracts against acidogenicity, marked by a tremendous reduction in the initial and final rate of the pH. This reduction in acidic value of pH could be directly co-related to the inhibition of the bacterial glycolytic enzymes. Furthermore, since the final pH values in glycolytic pH-drop assay stand for acid tolerance [28], the reduction in these values indicate the disruption of aciduracity potential as well. *S. mutans* by fact, is an aciduric bacteria which can carry out glycolysis at extremely low pH values. This is because the bacteria are able to maintain pH homeostasis across the cell membrane, which maintain the cytoplasmic alkalinity [37]. This aciduracity is mainly attributed by F- ATPase proton pump by maintaining pH gradient across the membrane [37]. The reduction in F- ATPase proton pump function by CR extract and ME fraction is evident from the data obtained. Thus, its suppression may contribute to rise in cytoplasmic acidity resulting in decreased acid adaptation. Cytoplasmic acidity will therefore lead to the impairment of functioning of a series of enzymes involved in physiological processes like glycolysis, cell persistence, IPS and EPS production, resulting in potential mortal effect on *S. mutans* [25].

The inhibitory role of the extracts (CR and ME) against Ag I/II or SpaP showed a moderate suppression of protein expression. As this surface antigen is known to critically induce bacterial adhesion [3], its reduction could be an alternate approach to combat dental caries.

The CLSM and scanning electron micrographs demonstrated the difference in architecture of the biofilm in control and treated samples. The apparent scattering of cells in treated samples can be related to the inhibition of glucan synthesis. This observation was further supported by the scanning electron microscopy. Unlike the untreated cells, entrenched in the sticky glucan pool, the treated cells had only traces of glucan resulting in cell dispersion and reduced cell to cell signalling, consequently obliterating the structural integrity of a biofilm. The reduction in the glucan synthesis however also confirmed the inhibition of GTFs activity.

Additionally, the expression profile showed that entire set of virulent genes were down regulated in the presence of crude extract and methanolic fraction. Gene *relA,* is known to be involved in the oxidative stress and acid tolerance mechanisms of *S. mutans* [38] whereas, *brpA* plays a critical role in formation of biofilm and its structural integrity. The repression of these genes will therefore lead to impaired acid tolerance and major structural defects in biofilm formation and integrity respectively, resulting in despaired virulence expressions. In addition, *gtfC*, catalyse the synthesis of water soluble and water insoluble glucan from sucrose. The reduction in the expression of this gene will thereby suppress the cascades involved in biofilm formation, cell wall integrity, adhesion promotion and surface biogenesis. The regulatory gene *comDE,* is a part of the quorum-sensing cascade of *S. mutans* [38]. The downregulation of this gene will attenuate the internal communication system utilized by the bacteria to alter their gene expression at a critical cell density, which may even lead to cell death.

Hence, the overall effect of CR extract and ME fraction is evidently anticariogenic as shown by *in vitro* studies. However, the complexity of the bacteria-host interaction leads us to the use of animal models, where caries lesions can be studied under controlled conditions. *In vivo* studies demonstrated that the daily topical exposure these extracts dramatically affected the ability of *S. mutans* to colonize on the tooth surfaces, consequently inhibiting the development of smooth surface caries and, to a lesser extent, sulcal surface carious lesions. The results obtained specify that the active compounds are available at efficacious concentrations in oral cavity despite of their brief exposure due to salivary clearance. It is known that the persistence of a therapeutic effect of topically applied agent in the oral cavity is a highly desirable in the development of chemotherapeutic approaches against dental caries [39]. The *in vivo* results (anticariogenic) may again be related to the effective inhibition of GTF activity and on bacterial glycolytic pathway which would reduce the pathogenicity of *S. mutans in vivo*. Our results are in harmony with a previous study which showed that a pronounced reduction in smooth-surface caries than the incidence of sulcal caries were observed in rats infected with *S. mutans* mutants defective in the production of either one or both Gtfs [40]. The *in vivo* effects of these extracts (CR and ME) are also validated with scanning electron micrographs and digital radiographs evidently showing reduced demineralization and a healthy pulp without any sequelae of dental caries (pulpitis). However, certain limitations can be overcome in the future studies that include the purification of pure compounds from the extracts. Also, instead of studying only *S. mutans* (monospecies), certain other oral micro- organisms (like *A. viscosus, L. acidophilus, S. salivarius*, *C. albicans, L. lactis*) that play a significant role in the prevalence of dental caries can also be studied.

However, as compared to our previous study which evaluated the potential of *Emblica officinalis* against cariogenic properties of *S. mutans* [3], the present study bears an upper hand. All the cariogenic factors that were evaluated in both these studies were evidently more reduced by *Z.officinale* than *E. officinalis*. Additionally, this study proves to be novel over the previous study [3], as it is extended to *in vivo* studies validating the *in vitro* results that showed *Z. officinale* to be a potential anticariogenic agent.

Hence, the study clearly indicates that CR extract and ME fraction of *Z. officinale* possess immense cariostatic potential. Furthermore, its non-toxic nature makes it more alluring for the development of novel anti-caries therapeutic agent.

## Conclusion

This study has investigated the effect of crude extract and methanolic fraction of Z*. officinale* against virulence properties of *S. mutans*. It reflects a prospective role of *Z. officinale* as a potential therapeutic agent against virulence traits of *S. mutans*. Hence, it can be a promising prophylactic therapeutic agent for dental caries.

## Additional files

Additional file 1:
**Nucleotide sequences of primers used in this study.**
Additional file 2:
**List of ligands identified from** ***Z. officinalis*** **Crude extract and Ethanolic fraction using GC-MS analysis.**
Additional file 3:
**Recovery of** ***Streptococcus mutans*** **on the following weeks after inoculation (×10** ^**4**^ **CFU).**
