# Supplementary material for: Inhibitory effect of zingiber officinale towards Streptococcus mutans virulence and caries development: in vitro and in vivo studies
Source: BMC Microbiol. 2015 Jan 16;15(1):1. doi: 10.1186/s12866-014-0320-5 (PMC4316655; doi:10.1186/s12866-014-0320-5)
Supplement: Additional file 1: — Nucleotide sequences of primers used in this study. [file 12866_2014_320_MOESM1_ESM.pdf]

Additional File 1: Nucleotide sequences of primers used in this study.

Genes	Description	Primer sequence (5' – 3')	
		**Forward**	**Reverse**
relA	Guanosine tetra (penta)-phosphatesynthetase	ACAAAAAGGGTATCGTCCGTACAT	AATCACGCTTGGTATTGCTAATTG
brpA	Biofilm-regulatory protein	GGAGGAGCTGCATCAGGATTC	AACTCCAGCACATCCAGCAAG
gtfC	Water soluble and insoluble glucan production	GGTTTAACGTCAAAATTAGCTGTATTAGC	CTCAACCAACCGCCACTGTT
comDE	Competence-stimulating peptide	ACAATTCCTTGAGTTCCATCCAAG	TGGTCTGCTGCCTGTTGC
