# Supplementary material for: Inhibitory effect of zingiber officinale towards Streptococcus mutans virulence and caries development: in vitro and in vivo studies
Source: BMC Microbiol. 2015 Jan 16;15(1):1. doi: 10.1186/s12866-014-0320-5 (PMC4316655; doi:10.1186/s12866-014-0320-5)
Supplement: Additional file 2: — List of ligands identified from Z. officinalis Crude extract and Ethanolic fraction using GC-MS analysis. [file 12866_2014_320_MOESM2_ESM.pdf]

Additional File 2: List of ligands identified from *Z. officinalis* using GC-MS analysis.

S. No	Compounds in crude fraction	Retention time
1	Decanaldehyde	8.685
2	2-Hendecanone	10.181
3	Citronellyl acetate	11.080
4	beta-(Z)-farnesene	11.176
5	alpha-terpineol	11.611
6	Isoborneol	11.693
7	N-Allyl-2-pyridone	12.021
8	Naphthalene	12.256
9	alpha-farnesene	12.379
10	Ocimenyl acetate	12.443
11	Sesquiphellandrene	12.753
12	Geraniol	13.626
13	Succinic anhydride	15.029
14	trans-Nerolidol	15.708
15	Ledenoxid-(ii)	16.011
16	beta Nerolidol	16.165
17	cis-.alpha.-Farnesene	16.602
18	Cyclohexanemethanol	16.757
19	Ledol	16.975
20	Ganaxolone	17.138
21	beta-eudesmol	17.634
22	Bisabolene	17.920
23	4-vinylguaiacol	18.193
24	Bisabolol	18.394
25	alpha-eudesmol	18.513
26	beta-Selinenol	18.630
27	Isoaromadendrene epoxide	18.851
28	Dehydrolinalool	19.078
29	Shogoal	19.323
30	Butylated Hydroxytoluene	20.009
31	Alpha-Curcumene	20.504
32	D-nerolidol	21.129
33	2-Decenylsuccinic anhydride	21.444
34	Citronellal	21.658
35	Methyl linolelaidate	21.783
36	Dodecanoic acid	22.093
37	Geranyl linalool	23.613
38	Carveol	24.065
39	trans-Nerolidol	24.504
40	Paradol	25.651
41	Tran- 10 - Shogaol	26.775
42	Octadecadienoic acid	27.866
43	Palmitoleic acid	28.987
44	Palmitic acid	29.428
45	Methyl linoleate	31.699
46	Zingerone	33.475
47	cis-Oleic Acid	38.235
48	Octadecanoic acid	41.025
49	Gingerol	46.483
50	Capsaicin	47.374

**S. No**	**Compounds in methanolic fraction**	**Retention time**
1	Methyl 2-oxopropanoate	4.618
2	Pentoxone	5.150
3	3-Hydroxybutan-2-one	5.766
4	Propanoic acid	6.140
5	Diacetone alcohol	6.526
6	Decanaldehyde	7.953
7	Butandiol	9.152
8	2,3-Butylene glycol	9.686
9	1,3-Cyclopentenedione	10.053
10	Butyrolactone	10.772
11	2-Furanmethanol	11.007
12	Zingiberene	12.039
13	2-Methylcyclopentanone	12.716
14	Paradol	13.944
15	Furaneol	16.141
16	2,4-Hexanedione	17.245
17	α Curcumen	17.967
18	p-Vinylguaiacol	18.194
19	Tran- 10 - Shogaol	19.118
20	D-nerolidol	20.385
21	Cis -6-shagaol	20.726
22	1,E-11,Z-13-Octadecatriene	21.428
23	5-Hydrxoymethylfurfural	21.550
24	Cyclopropaneoctanoic acid	21.783
25	Zingerone	22.290
26	Methyl pyroglutamate	22.809
27	Carveol	24.506
28	N-Allyl-2-pyridone	25.368
29	Palmitate	29.483
30	Secoisolariciresinol	30.689
31	Elaidic acid	38.160
32	Alpha tocopherol	39.602
33	Methyl linoleate	41.009
