# Supplementary material for: Inhibitory effect of zingiber officinale towards Streptococcus mutans virulence and caries development: in vitro and in vivo studies
Source: BMC Microbiol. 2015 Jan 16;15(1):1. doi: 10.1186/s12866-014-0320-5 (PMC4316655; doi:10.1186/s12866-014-0320-5)
Supplement: Additional file 3: — Recovery of Streptococcus mutans on the following weeks after inoculation (×10 4 CFU). [file 12866_2014_320_MOESM3_ESM.pdf]

Additional File 3: Recovery of *Streptococcus mutans* on the following weeks after inoculation ($\times 10^4$ CFU).

Weeks	Control	Crude extract	Methanolic fraction
**0**	0	0	0
**1**	118.45 ± 4.54	124.23 ± 4.77	123.45 ± 4.56
**2**	123.04 ± 3.89	109.45 ± 3.99	118.34 ± 7.67
**3**	131.78 ± 7.89	102.26 ± 7.07	111.34 ± 5.44
**4**	145.27 ± 8.11	95.23 ± 6.22	102.23 ± 6.24
**5**	168.67 ± 5.46	85.10 ± 5.22	98.11 ± 5.24
